# The Insanity of Jealousy

**Published:** 1890-11

**Authors:** B. Ball

**Affiliations:** Paris


### THE INSANITY OF JEALOUSY.

PROFESSOR B. BALL, PARIS.

WITH REMARKS BY S. L., BULLETIN MÉDICAL 57, '90.

" Eifersucht ist eine Leidenschaft, die mit Eifersucht was Leidenschaft " (Jealousy is a passion which zealously tries to create trouble).

Under jealousy one can understand a general tendency similar to envy and, like it, founded on egotism. It is generally thought that jealousy from love carries within itself its excuse, being only an excess of the most tender sentiment, hence juries are very apt to be lenient to those who committed crimes while under the yoke of this passion, while in fact jealousy is only a very pronounced egotism, as the patient finds himself wounded far more in his self-esteem than in his affections. A few examples will explain my meaning. Here we have a well-educated woman of forty-six years, with the traces of former beauty still on her face, and who became jealous on account of her age, and we know climaxis is always a tender spot with women. She was the head cook in a large establishment, and she prided herself on her excellent taste, considering herself the queen of the table, when, without cause, she became suspicious of her husband, and bothered him constantly in such a manner that he thought to have her confined in an asylum, but loving her still, he sacrificed himself and shot himself in her presence. She never lost a tear, she rather felt pleased, and ate and slept as well as ever. This indifference to persons near and dear to one is characteristic of the insane, who are indifferent to everything which is foreign to their fixed idea, and this woman feels pleased that her husband cannot cheat her any more.

In other cases, as our second one, the wife may not love her husband. This patient, the baby of a large family, was spoiled in her infancy and every wish gratified, so that she considered herself its centerpoint. Unfortunately she married a man who gave way to all her whims. They had two children, one died from meningitis, and during her second pregnancy she suffered from excessive jealousy. Her husband, employed on the marine staff, was obliged to be frequently from home, but she followed him everywhere and accused him of having intimate relations with other women and raised mischief even in the street, so that she fell into the hands of the police, and was declared insane. At the asylum she tried to breed discontent, broke everything she could lay her hands on, was full of halluciations of hearing the conversations of her husband with these women. She is now demented and incurable.

Dr. M. P. Moreau proposed the following classification: 1. Feeble jealousy, constant cavil about trifles. 2. Quarrels and fights caused by jealousy, threatening to kill its object, but does not accomplish it. 3. Violent jealousy, murder. 4. Murder followed by suicide. May it not be better to classify it only as simple jealousy and jealousy with alienation and with or without halluciation? Jealous mothers have killed their children and then committed suicide (Medea and Jason). Heredity and education play here also a great part; though women are more given to jealousy, still one meets men who are perfect tyrants in their households. Accidental diseases may also provoke jealousy, and sometimes it originates in a traumatism especially of the head or from diseases of the skull affecting the brain. Zymotic diseases, chlorosis, anæmia, hysteria, but especially alcoholism may often be blamed for these different degrees of jealousy, and the men are often jealous after drinking. Pregnancy and menopause may lead to insane delusions of jealousy, from simple melancholy to folie circulaire and hysterical insanity is well known. Some cases are curable, but the prognosis is bad, when deliria of persecution are mostly found in patients born with a degenerated brain, and sequestration is the only means to calm such turbulent brains. Such spoiled children, spoiled even after being grown up, must learn the moral power of a superior force, of inflexible rules which they must obey.

Homœopathy offers great resources in these cases, and still even our remedies will act better when such a patient is removed from home influences, where every trifle goads the patient on to fresh exhibition of their pranks. It is a great gain, as Hyatt teaches, when such a patient learns that obedience to the rules is the first requisite, and as their strength returns, the cloud will gradually melt away. In the third edition of my *Therapeutics*, page 381, we give some well-tried drugs. Who would not think of Apis or Lachesis in cases originating during puerperium or menopause? Some writers even called Apis "the widow's remedy," on account of its sexual irritability and thus it may be suitable to light as well as to severe cases.

Weary at heart is characteristic of Lachesis, and weary of life such a patient doubts everything, and thus also the fidelity of her husband, and hence homicidal and suicidal ideas enter the diseased mind. A dose of Lachesis might have restored the troubled conscience of a Medea. The differential points between Lachesis and Naja await yet to be worked out, and Naja is too much neglected in our studies of mental diseases.

Ischæmia of the brain hints to Arsenicum, worn out mentally and bodily, such patients look back to a happy past, fearing a desolate future and envy and jealousy are the natural consequences, hence constant anguish, for in her hallucinations she sees others enjoy the pleasures belonging to her and in her hopelessness the suicide by the cord offers the only relief. Religious melancholia is no idle dream, and the woman may be called blessed where this consolation offers her some substitute for the imagined wrongs of her love and affection. What a difference between the impatience and restlessness so characteristic of Arsenicum and the calm resignation so pointedly manifested in Ignatia. Richard Hughes is right when he says that Ignatia can only be indicated at the very beginning of epilepsy or any other nervous disease, and thus also it suits only Moreau's first type of jealousy; it is yet only an inward grief, and she tries to hide her mental agony from those she loves so well as also from the outside world. Though we meet the same great sadness from disappointed affection in Natrum-muriaticum, still it suits better a more advanced state of jealousy; she is fretful, rejects consolation and grieves that she has lost some of the attractiveness which is so much a woman's greatest pride. In Natrum-muriaticum and in that third degree of Moreau, which may indicate Arsenicum, the vital power is below par, there are bodily ailments, perhaps latent still, but undermining the health of the sufferer, while in those cases where Aurum suits, the patient usually has nothing to complain of in relation to health and she feels, therefore, so much more mortified that somebody else stands higher in the affection of her lover. Thus she loses all confidence in herself, and either seeks consolation in the Church or homicidal and suicidal ideas render her a dangerous patient. It is really often hard to decide whether Arsenicum or Aurum is more suitable and the anamnesis may be necessary to decide it. The Arsenicum patient is not in as good health generally as the Aurum patient, when the mental disorder is the chief disease, though hereditary syphilis may in some cases be the starting-point. In many cases where Professor Ball gives his purgatives, we can do better service with our Natrum-muriaticum or Sulphuricum, or with our preparation of Gold.

In Moreau's highest degree, that jealousy with outspoken mental alienation, he introduces to us these *infants terribles,* these spoiled children whose egotism become the bane of their life. It may not yet be too late for antipsoric treatment, and the Salts of Lime and Sulphur, at long intervals, may aid us in eradicating this bad disposition. We meet in Sulphur that childish peevishness in grown persons, that egotism in her own perfection which radiates upon all who surround her, and, when not acknowledged, render her actions unbearable and mischievous. How strongly the somatic as well as physical pruritus, found partially in Sulphur, reminds one of Hyoscyamus and Stramonium! There is hardly any remedy which has such ungovernable rage as the Henbane, and no wonder that Hyoscyamus became a favorite in the asylums under old-school management. Lascivious jealousy is our characteristic for Hyoscyamus, while hallucinatory jealous alienation often finds its counterpart in Stramonium, a grand remedy where hysteria prevails in the patient or in her family.

It would lead me too far to mention more remedies for this insane jealousy; in fact, any remedy which covers the totality of the case may be indicated, but let us not forget the sage advice of the French alienist that craziness *per se* is already a proof of minus in the vital force, that most lunatics are qualitatively anæmic, and that they must be treated like children, with firmness and kindness combined, which is only possible in closed institutions, where the aggravations from home influence can be averted. Let us call them hospitals for mental affections and the stigma of an asylum will not be dreaded so much as it is at present the case, and the earlier the troubled mind comes at rest, the greater the chance for full recovery.

